# ﻿Reinstatement of *Struthanthusvenetus* (Loranthaceae): an endemic species of central Mexico

**DOI:** 10.3897/phytokeys.249.127215

**Published:** 2024-11-14

**Authors:** Maria Guadalupe Maldonado-Borja, Mónica Isabel Miguel-Vázquez, Adolfo Espejo-Serna, Rosa Cerros-Tlatilpa

**Affiliations:** 1 Maestría en Manejo de Recursos Naturales, Centro de Investigaciones Biológicas, Universidad Autónoma del Estado de Morelos, Av. Universidad 1001, Col. Chamilpa, Cuernavaca, Morelos, 62210, Mexico; 2 Facultad de Ciencias, Universidad Autónoma de San Luis Potosí, Av. Chapultepec 1570, Priv. del Pedregal, San Luis Potosí, S.L.P., 78295, Mexico; 3 Herbario Metropolitano, Departamento de Biología, División de Ciencias Biológicas y de la Salud, Universidad Autónoma Metropolitana-Iztapalapa, Iztapalapa, Ciudad de México 09340, Mexico; 4 Facultad de Ciencias Biológicas, Universidad Autónoma del Estado de Morelos, Av. Universidad 1001, Col. Chamilpa, Cuernavaca, Morelos, 62210, Mexico

**Keywords:** Dioecious, epitype, hemiparasitic, mistletoe, nomenclature, Psittacanthinae, taxonomy

## Abstract

*Struthanthus* Mart. is a challenging genus because the abundance of reproductively incomplete specimens (lacking mature pistillate and staminate flowers and fruit) has inspired the description of many species, resulting in a long list of names to be verified. In addition, the genus currently lacks a monographic treatment. *Struthanthusvenetus* (Kunth) Blume was previously considered a heterotypic synonym of *S.interruptus* (Kunth) G. Don. However, examination of herbarium specimens and observation of living plants demonstrate that *S.venetus* is a distinct species distinguishable from *S.interruptus*. Here, we propose the reinstatement of *S.venetus* along with a complete description of the species and the designation of an epitype to complement the reproductively incomplete (lacking flowers) and severely fragmented type.

## ﻿Introduction

*Struthanthus* Mart. (Martius 1830) is a neotropical genus of mistletoe belonging to the tribe Psittacantheae subtribe Psittacanthinae in Loranthaceae (Nickrent et al. 2010; Kuijt 2012; Nickrent et al. 2019). Being closely related to *Cladocolea* Tiegh. and *Peristethium* Tiegh., members of *Struthanthus* are mainly dioecious and consist of approximately 60 to 70 species (Kuijt 1981; Vidal-Russell and Nickrent 2008; Kuijt 2012; Robles et al. 2016; Galván-González 2016; Grímsson et al. 2018; Caires and Dettke 2020). A key requisite for the identification of *Struthanthus* is the presence of flowers, especially staminate ones. Taxonomic studies in *Struthanthus* have been challenging because the genus currently lacks a monographic treatment (Dettke and Waechter 2012; Kuijt 2016; Robles et al. 2016, Maldonado-Borja et al. 2023). In addition, the genus contains a long list of synonyms and many taxa that were described from fruiting individuals or from specimens containing only pistillate or staminate inflorescences, these sometimes at the bud stage (Kuijt 2016; Caires and Dettke 2020). Moreover, herbarium specimens of putative species of *Struthanthus* are often sterile, which causes uncertainties for classification at the generic and species levels.

In Mexico, 15 species of *Struthanthus* are currently accepted (Maldonado-Borja et al. 2023), and dozens more have been proposed. One of these is *S.venetus* (Kunth) Blume, a species that has been treated as one of the eight to 11 synonyms of *S.interruptus* (Kunth) G.Don (Kuijt 1980, Kuijt 2009; Kuijt 2016; Tropicos.org 2024; POWO 2024). Both *S.venetus* and *S.interruptus* were described by Kunth (Humboldt et al. 1818 [1820]) based on an incomplete specimen that has obscured the proper identification of the species. Therefore, this paper seeks to reestablish *S.venetus*, providing evidence from morphological characters and germination of pollen grains to distinguish it from similar species from Mexico, particularly *S.interruptus*.

## ﻿Methods

### ﻿Morphological analysis

The study is based on specimens from the herbaria CIIDIR, ENCB, HUAP, HUMO, IEB, MEXU, UAMIZ, and XAL (acronyms following Thiers 2024). Digitalized images from COLO, DES, F, GH, K, MA, MO, NY, P, RSA, SMU, U, UC, US, and VT were also studied. Morphological characters were analyzed from field-collected plants. We encountered several flowering individuals in March 2022, and then proceeded to carry out fieldwork from May 2022 to October 2023 to collect specimens with flowers and fruits. Comparisons between fresh and dried morphological characters were performed with a stereomicroscope using specimens collected from the states of Ciudad de México, Estado de México, Guanajuato, Guerrero, Morelos, and Puebla.

Relevant literature was reviewed, as well as the protologues of all taxa involved in this study. Nomenclatural decisions were based on the rules and recommendations provided in the International Code of Nomenclature for algae, fungi, and plants (ICBN; Turland et al. 2018). A distribution map was generated with QGIS (QGIS Development Team 2024; version 3.26.2) with coordinates based on data from fieldwork, herbarium specimens, and observations obtained from iNaturalist (2024).

### ﻿Fluorescence microscopy

Flowers of *S.venetus* were collected in central Mexico from March 2022 to October 2023 and consisted of 43 accessions at different stages of anthesis. In addition, fresh inflorescences and flowers at different stages were collected in the field and fixed in FPA (1:1:18 37% formaldehyde: propionic acid: 70% ethanol). Flowers from each specimen were examined to verify the presence of fully developed reproductive structures. Pollen viability was assessed through a germination test by fluorescence microscopy to determine if *S.venetus* accessions had functionally bisexual flowers. This analysis was carried out at the Laboratorio de Desarrollo en Plantas in the Facultad de Ciencias, Universidad Nacional Autónoma de México (UNAM), in August 2023. The pistils of flowers were removed and washed in distilled water followed by a wash in sodium hydroxide (NaOH 1N) for four days to soften the pistil tissue. The styles were rinsed in distilled water and softly macerated for five minutes on a slide with 1 or 2 drops of cotton blue pigment from aniline at 0.1% in 0.1 N potassium phosphate (O’Brien and McCully 1981, Torres and Murashige 1985). The stigma and style were observed under a fluorescence microscope (Olympus Provis AX750) searching for pollen tubes. Cotton blue has a great affinity to callose, allowing identification of the pollen tubes (Martin 1959), and pollen grains that emitted a bright green tube were considered to have germinated.

## ﻿Results

**Figure 1.**
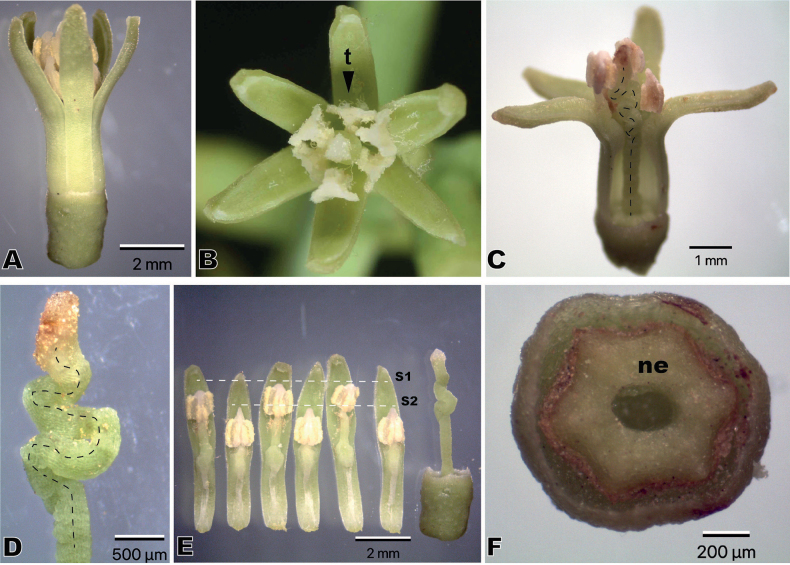
Flower morphology of *Struthanthusvenetus* (Loranthaceae) **A** lateral view of flower at early anthesis **B** upper view of flower at full anthesis showing ascendant petals, the pistil with stigma in the center, and functional stamens with tufts of trichomes (t) behind the anthers **C** lateral view of flower at full anthesis with a petal removed to show the contour of the convolute style (black dashed line) **D** lateral view of the convolute style with brownish stigma **E** long (S1) and short (S2) series of stamens next to the whole pistil (right side) **F** hexagonal nectary (ne) at the base of the style (central circular scar).

A total of 102 specimens of *S.venetus* were examined, 59 from herbaria and 43 collected during fieldwork. Forty percent of these specimens have only flowers, whilst 34% have only fruit, 10% are with both flowers and fruit, 10% possess flower buds, and 6% are sterile. *Struthanthusvenetus* is a monoclinal species whose flowers reach 6–8 mm in length at full anthesis. Flowers are formed by six unfused petals, valvate, and green to yellowish in color; six well-developed stamens in two alternating series (three long and three short, all with viable pollen); and a pistil composed of an inferior ovary, a convolute style that is the same length of the first stamen series ending in a differentiated stigma, and a thick hexagonal ring-shaped nectary (Fig. 1A–F).

**Figure 2.**
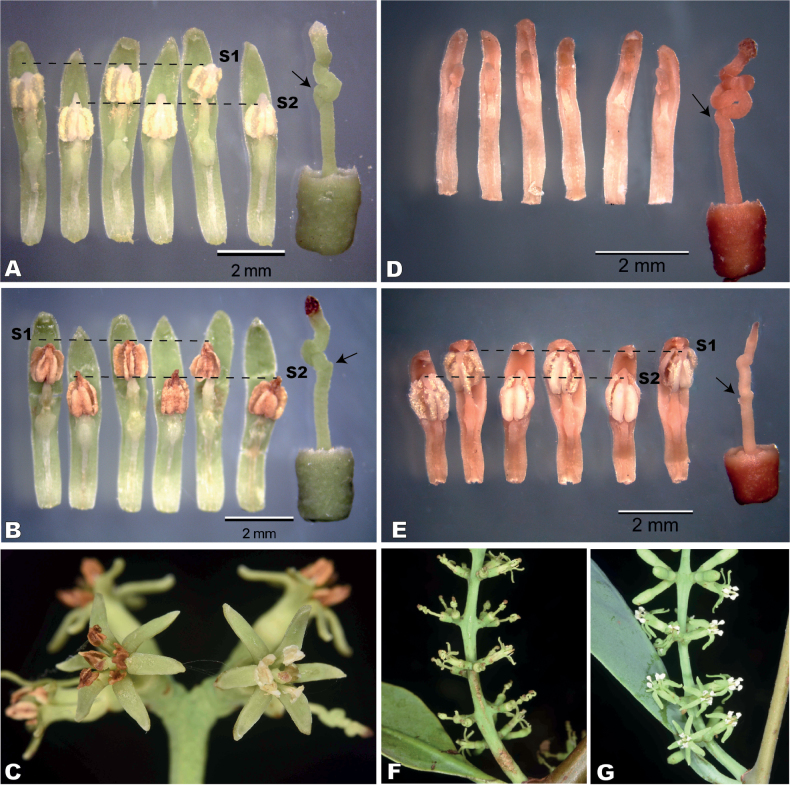
Comparative reproductive morphology between *Struthanthusvenetus* and *S.interruptus* (Loranthaceae). *S.venetus*: **A** dissected flower at early anthesis. Note that all stamens are well-developed and arranged in two alternating series (S1 and S2). Pistil showing the convoluted style at right **B** dissected flower during late anthesis **C** inflorescence with flowers in early and late anthesis. *S.interruptus*: **D** dissected pistillate flower showing vestigial, non-functional staminodes and pistil with convoluted style **E** dissected petals of a staminate flower showing two series of alternating stamens with pollen grains visible and an undifferentiated, wavy pistillode **F** pistillate inflorescence showing some flowers without petals **G** staminate inflorescence with flowers at full anthesis exhibiting bright white anthers. Black arrows in pistils are pointing to the first convolution in styles.

Our observations indicate that flowers of *S.venetus* at early and late anthesis display male and female organs that are functional and well-developed (Fig. 2A–C). Stamens in early anthesis have white anthers with yellowish pollen, whereas in late anthesis anthers become brownish with few pollen grains. Interestingly, inflorescences contain flowers at different stages of maturity (Fig. 2C). On the other hand, *S.interruptus* has clearly unisexual flowers (Fig. 2D–E) with vestigial organs of the opposite sex. Pistillate flowers have a convolute style with staminodes poorly distinguishable (Fig. 2D), while male flowers show a wavy style and well-developed anthers with pollen.

**Figure 3.**
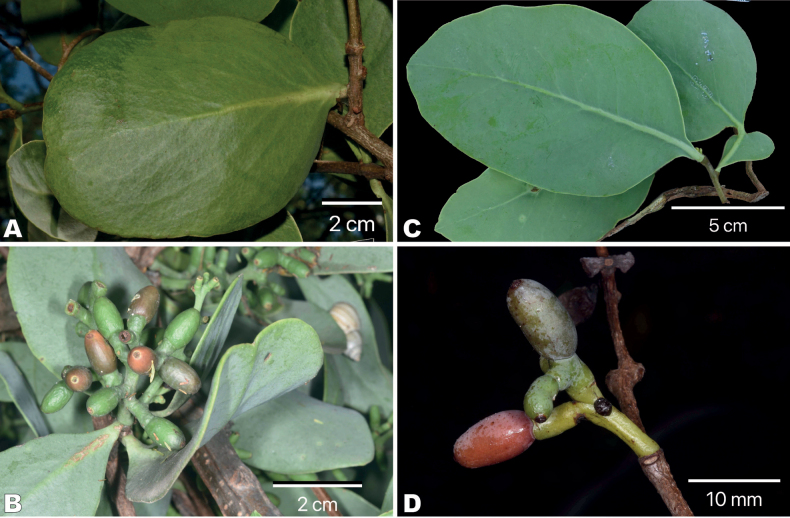
Leaves and fruits of *Struthanthusvenetus* and *S.interruptus* (Loranthaceae). *S.venetus*: **A** leaves **B** infructescence with mature and unmatured fruits. *S.interruptus*: **C** leaves **D** infructescence, fruit reddish, glaucous.

Regarding non-reproductive morphological characters, leaves of *S.venetus* are green and crasso-coriaceous (Fig. 3A) and fruit are ovoid to subglobose, granulated, and rusty colored when ripe, 4.0–6.0 × 6.5–10.0 mm (Fig. 3B). This contrasts with *S.interruptus*, a species with bluish-green tinge and papery or chartaceous leaves (Fig. 3C) and ovoid to ellipsoid smooth fruit that ripen orange-reddish, 6.6–7.2 × 10.8–12.8 mm (Fig. 3D).

**Figure 4.**
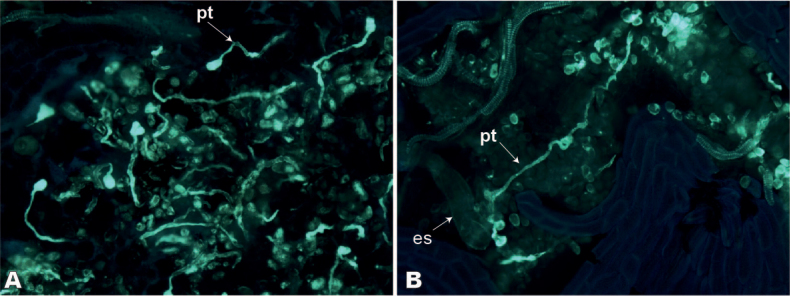
Fluorescence microscopy showing germinated pollen grains and pollen tubes of *Struthanthusvenetus* (Loranthaceae) flowers at full anthesis **A** pollen grains germinating and growing pollen tubes (pt) into the stigma **B** pollen tubes (pt) growing through the style to reach the embryo sac (es). Reference specimen: *M. I. Miguel V. 1300* (HUMO, accession number 40028).

Flowers at full anthesis of *S.venetus* examined using fluorescence microscopy confirmed that pollen grains were viable, capable of germinating on a receptive stigma and developing a specialized tube up to two-thirds the length of the style tissue as a conduit to the embryo sac (Fig. 4A, B).

### ﻿Epitypification

**Figure 5.**
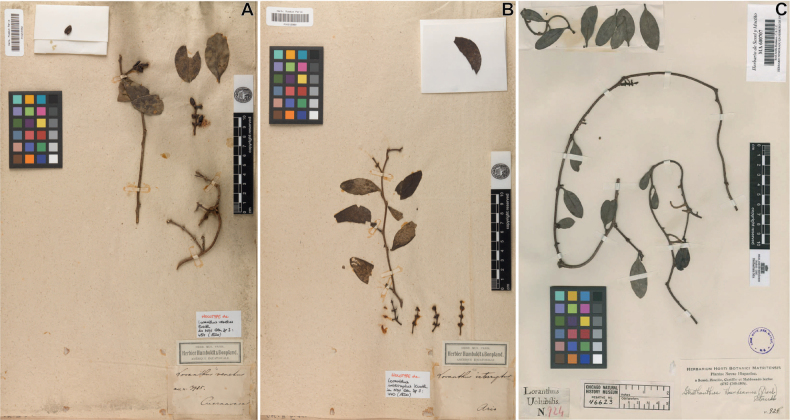
Types of *Struthanthusvenetus*, *S.volubilis*, and *S.interruptus* (Loranthaceae) **A** holotype of *Loranthusvenetus* (≡ *Struthanthusvenetus*), *Humboldt & Bonpland 3985* (P-00215992!) **B** holotype of *S.interruptus*, *Humboldt & Bonpland s.n*. (P-00215992!) **C** lectotype of *L.volubilis* (≡ *S.volubilis*), *Sessé 924* (MA-600707!).

In 1820, Karl S. Kunth (Humboldt et al. 1818 [1820]) described *Loranthusvenetus* Kunth based on the specimen *Humboldt & Bonpland 3985* (P-00215992!; Fig. 5A), which was collected during the expedition to New Spain by Alexander von Humboldt & Aimé Bonpland between 1799 to 1804 (Humboldt 1802–1804, 1827; Humboldt et al. 1818 [1820]; Stoddard 1859; Minguet 1969; Labastida 2016). According to Minguet (1969), Humboldt and Bonpland visited Cuernavaca between April 9^th^ and 11^th^, 1803; hence, the collection of *L.venetus* was made during those dates, at an elevation of 1,555 m (“altitudo 850 hexapedalis”, from the protologue).

The holotype of *L.venetus* only contain leaves and infructescences (Fig. 5A), and the species diagnosis prepared by Kunth (Humboldt et al. 1818 [1820]) reads as follow: “*foliis elliptico oblongis aut abovatis, apice rotundatis, crasso-coriaceis, glaucescentibus*…”. Because of its elliptic-oblong or ovate leaves, Kunth suggested that *L.venetus* was closely related to *L.ovalifolius* Ruiz & Pav. (= *Oryctanthusovalifolius* (Ruiz & Pav.) J.F.Macbr.), which is illustrated in plate CCLXXVIIb by Ruiz and Pavón (1802). This plate depicts leaf blades whose shape is consistent in both young and mature leaves. However, *S.venetus* shows heterophylly, with leaf blades varying in size and shape on the same individual along a branch. In addition, although the diagnosis for *S.venetus* mentions that the species blooms in April (“*Floret Aprili*”), Kunth mentioned that flowers were not seen (“Flores haud vidi”; Humboldt et al. 1818 [1820]). Given that most species in *Struthanthus* are dioecious and the flowers of *L.venetus* were not examined when preparing its protologue, it was assumed that the species was dioecious.

In 1830, Blume transferred *L.venetus* Kunth along with 35 more taxa to the genus *Struthanthus*. In this publication there is no association between the generic name and the epithet, where only the basionyms were listed. However, this is a valid publication following article 41.3 of the ICBN (Turland et al. 2018): “Before 1 January 1953 an indirect reference to a basionym or replaced synonym is sufficient for valid publication of a new combination, name at new rank, or replacement name.” Therefore, *S.venetus* (Kunth) Blume is a valid name, along with the other species of *Struthanthus* that Blume transferred in the same work.

*Struthanthusvenetus* was cited for the first time in the book *Trees and Shrubs of Mexico* by Standley (1922), where a diagnosis for the genus was prepared to include the Mexican species. Standley mentioned that the type of *S.venetus* was from Cuernavaca, Morelos and that *L.volubilis* Sessé & Moc., described later in 1888, could represent the same taxon. *Loranthusvolubilis* was described from specimens collected in Cuernavaca (as “Cuahunahuacae”) during the Royal Botanical Expedition to New Spain (Sessé and Mociño 1888). The description of the taxon indicates voluble stems with opposite, rarely alternate, ovate, fleshy, glabrous, shortly petiolate leaves; inflorescences in axillary clusters with trifloral pedicels; white to greenish flowers; and oval fruits. In addition, the Náhuatl name *Teapizmictiquahuitl* is recorded for this species.

But which is the type for *L.volubilis*? A recurrent problem in the Sessé & Mociño specimens is that plants are not accompanied with data about location, date, collection number, and collector name. In 1997, Nelson designated the specimen *M. Sessé et al. s.n.* (MA-600707!; Cuatrec. No. 924; Negative/Types: F-46623) as the lectotype of *L.volubilis* (Fig. 5C). At the herbarium of the Real Jardín Botánico de Madrid (MA), there are two additional collections identified as *L.volubilis*: *M. Sessé et al. s.n.* (MA-606685!; 168-PB) and *M. Sessé & J.M. Mociño s.n.* (MA-606815!; Cuatrec. No. 4952), both determined by Standley (McVaugh 2000) as *S.haenkeanus* Standl. (≡ *S.haenkei* (Presl.) Engl.), which is a synonym of *S.interruptus*. An examination of the specimen *M. Sessé & J.M. Mociño 4952* reveals that its characters does not match the description of *L.volubilis* of having shortly petiolate leaves, with petioles on the specimen measuring 0.8–1.5 cm. On the other hand, the specimens *M. Sessé et al. 168-PB* and *M. Sessé et al. 924* have leaves with petioles of 4.0–6.0 mm and leaves with acute apex, which coincides with the description of *S.venetus* by Kunth (Humboldt et al. 1818 [1820]): “…*Folia opposita, breviter petiolata, elliptico-oblonga aut obovata, apice rotundata… Petioli 2 lineas longi*.” (translated as “Leaves opposite, shortly petiolate, elliptical-oblong or obovate, round apex… Petiole 4.2 mm long.”). This description mostly agrees with the one provided for *L.volubilis* by Sessé and Mociño (1888): “Folia opposita, raro alterna, ouata, carnosa, utrinque glabra, uenosa, breuiter peciolata.” (translated as “Leaves opposite, rarely alternate, ovate, fleshy, glabrous on both sides, veined, shortly petiolate”).

**Figure 6.**
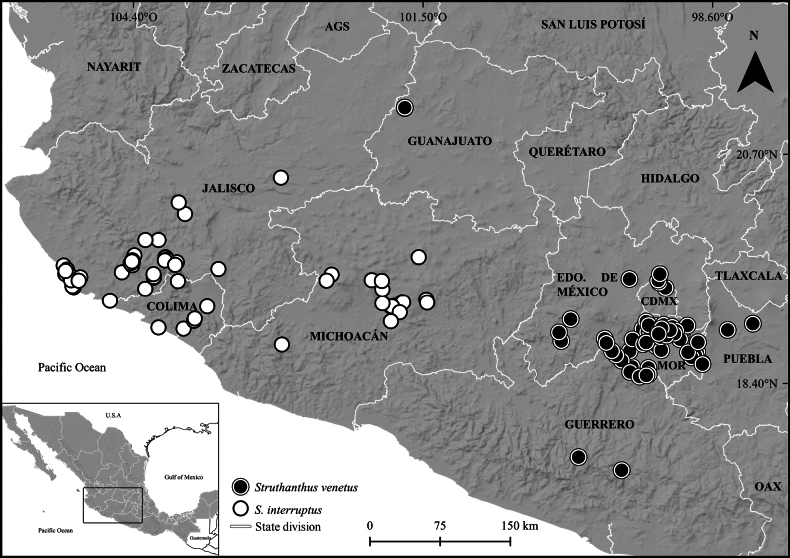
Distribution range of *Struthanthusinterruptus* (Loranthaceae; white circles) and *S.venetus* (black circles) in México. AGS= Aguascalientes, CDMX= Ciudad de México, EDO. DE MÉXICO= Estado de México, MOR= Morelos, OAX= Oaxaca.

The specimen *M. Sessé et al. s.n*. (MA-606685!; 168-PB) has flower buds while *M. Sessé et al. 924* has infructescences. The most likely explanation is that both specimens were collected in the second expedition conducted in Cuernavaca, Morelos, and other localities visited in Guerrero. Botanists began this expedition in March of 1789 and established their headquarters in Cuernavaca until December of the same year (García 2011). The fact that the two specimens collected during this expedition were either flowering or fruiting shows that explorers were present in the area for several months. All these botanical expeditions were carried out by Sessé et al. from 1787 to 1790 to New Spain and the localities of specimens collected in Cuernavaca match with the known distribution of *S.venetus* (Fig. 6) (Álvarez 1952, 1953; McVaugh 1969; Blanco Fernández de Caleya 1995; Maldonado 2000; Blanco Fernández de Caleya et al. 2010). According to the morphological comparison of the herbarium material, *L.volubilis* and *S.venetus* present similar morphological characters such as petiole length, leave and apex shape, and have the same distribution. For the above mentioned reasons, we are considering here *L.volubilis* to be conspecific with *S.venetus*.

*Struthanthusvenetus* is not a synonym of *S.interruptus*.

In 1975, Kuijt proposed *Struthanthushaenkei* (Presl.) Standl. as a synonym of *S.venetus*. In his study he examined 16 specimens, of which and to our understanding, only four correspond to *S.venetus*: *Bonpland 3985* (P00215992!), *Nagel 8026* (MEXU-103129!, MEXU-103130!, MEXU-103131!; GH 13259!), *Pringle 6185* (MEXU-11956!; UC-116705!; UVMVT-242343!; COLO-02212413!; US-930060!), and *Rose 8584a* (GH!). As for the other 12 cited specimens, *Brandegee s.n.* (♀ fr, UC-116703!) agrees with *S.condensatus* Kuijt because the inflorescences have two triads and a cymbiform bract that embraces the bracteoles. Also, it matches the distribution range reported for this taxon (Pacific coast of México). The two duplicate specimens of *Hinton 1281* (♀ fl, GH!; US-1636506!; DES-00025368!) match with *S.crassipes* (Oliv.) Eichler since the leaves have an acute apex and inflorescences are racemes with eight pairs of pedicellate triads and subsessile flowers. The *Lumholtz s.n.* (GH!) specimen is sterile, which makes uncertain its identification. The two specimens by *Rowell 2935* (SMU) and *Turner 2106* (SMU) were not located. The seven remaining material examined correspond to *S.interruptus*: *Gregory & Eiten 318* (♂ fl, NY!, SMU), *Haenke 146* (♀ fl, fr, MO!, P!, F!, GH!, PR), *King 1194* (♀ fl, UC!, NY!), *Leavenworth & Hoogstraal 1656* (MO!), *Moseed M-1398* (♂ fl, GH!), *Palmer 337* (♀ fr, UC!), and *Palmer 982* (♀ fr, UC!).

Five years later, Kuijt (1980) changed his opinion and treated *S.venetus* as synonym of *S.interruptus* (Kunth) Blume. The basonym of *S.venetus* is described before *S.interruptus*Humboldt et al. 1818 [1820]), and according to the principle of priority (Art. 11.3; Turland et al. 2018), *S.venetus* would have priority over *S.interruptus*. *Struthanthusinterruptus* and *S.venetus* were described based on incomplete material (types lacking flowers and severely fragmented), which has added confusion and misunderstanding for both species. It should be noted that Blume (1830) did not transfer *L.interruptus* to *Struthanthus*, but to *Spirostylis* Presl; in fact, it was G. Don in 1834 who transferred *L.interruptus* to *Struthanthus*.

**Figure 7.**
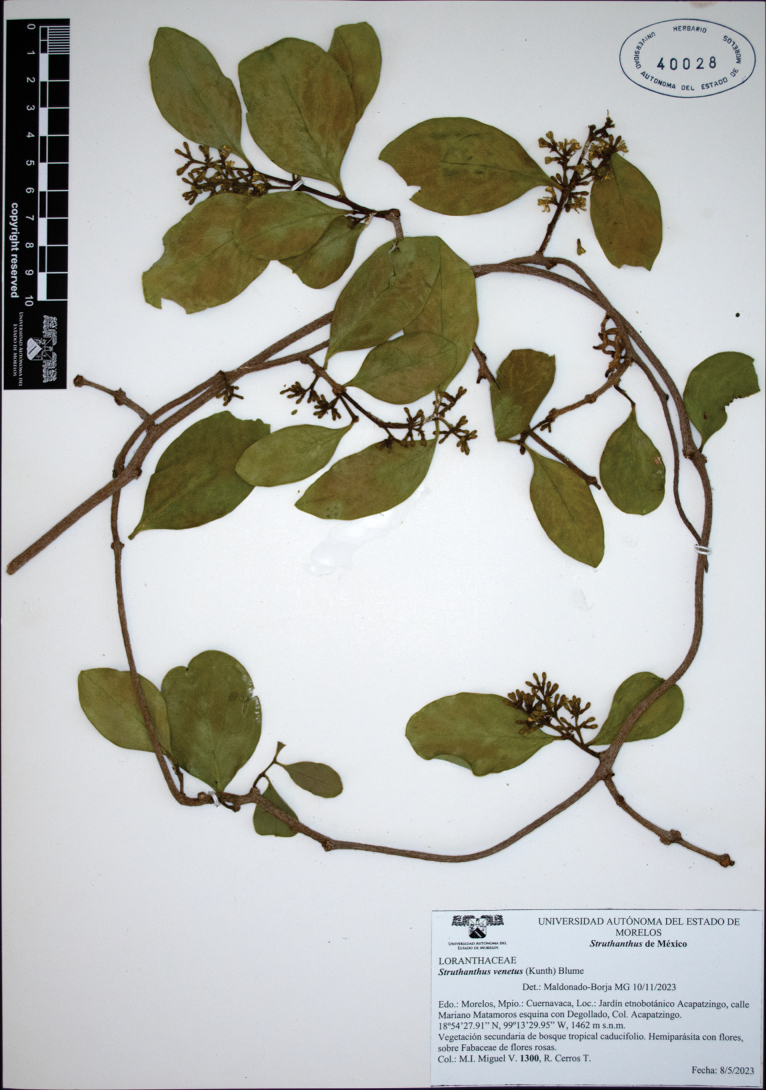
Epitype of *Loranthusvenetus* (≡ *Struthanthusvenetus*), *M.I. Miguel V. 1300* (HUMO-40028!); collected in Cuernavaca, Morelos at the type locality.

Therefore, as a measure to allow interpretation of the primary type of *L.venetus*, we propose an epitype, in which the inflorescences and reproductive characters (gynoecium and androecium) can be studied (Fig. 7). This action follows art. 9.9 of the International Code of Nomenclature for algae, fungi, and plants (Turland et al. 2018), as well as the recommendations of Lendemer (2020) and Sennikov (2022), which are: (1) the taxonomic link between the epitype and the protologue and the type it supports; (2) same location as the type; (3) explanation of why the type is ambiguous and justification of epitype; and (4) a statement that the designated epitype represents the same taxon as that to which the name currently applies.

An attempt was made to collect specimens of *S.venetus* growing on the same species or genus as the host recorded in the type collection (*Diospyrosnigra* (J.F. Gmel.) Perr., Ebenaceae). However, *Diospyros* was not found at the desired elevation, and the individuals found in the region were not being parasitized by *S.venetus*. Therefore, the material selected as epitype was collected at the type locality of Cuernavaca, Morelos, at an elevation comparable to the one recorded for the holotype.

### ﻿Taxonomic treatment

#### ﻿ Struthanthus venetus

Taxon classificationPlantaeSantalalesLoranthaceae

(Kunth) Blume, Syst. Veg. (ed. 15 bis) 7(2): 1731. 1830.

990BEB8B-D65A-51D9-A5DE-65E8185023F1

Fig. 1A–F

Loranthus venetus Kunth, Nov. Gen. Sp. (quarto ed.) 3: 434. 1818[1820].
Loranthus volubilis Sessé & Moc., Pl. Nov. Hisp.: 51. 1888. Type: Quahunahuacaearboribus [Cuernavaca, Morelos], *M. Sessé et al. s.n*. (lectotype, designated by Nelson 1997, pg. 401: MA-600707!; Cuatrec. No. 924; Neg./Types: F-46623).

##### Type material.

Mexico • Morelos: crescit in Regno Mexicano, prope Cuernavaca, altitudo 850 hexapedalis [1554.5 m], *Humboldt & Bonpland 3985* (holotype, photography in JSTOR: P-00215992!; Fig. 5A); (**epitype**, designated here; Fig. 7). Mexico • Morelos: Cuernavaca, Jardín etnobotánico Acapatzingo, calle Mariano Matamoros esquina con Degollado, col. Acapatzingo, 18°54'27.91"N, 99°13'29.95"W [18.9079155, -99.2243666], 1462 m, on *Gliricidiasepium* (Jacq.) Kunth (Fabaceae), 8 May 2023 (fl), *M.I. Miguel V. 1300* (HUMO-40028).

##### Description.

***Shrub*** hemiparasitic, perennial, woody, scandent, monoecious-monocline. ***Haustorium*** with terete epicortical roots, emerging from the base of the plant, secondary haustoria. ***Stems*** terete, glabrous, glaucous when young, brown to brown-grayish with lenticels; green, voluble with nodes flat when young; with epicortical roots. ***Leaves*** opposite, subopposite or alternate, decussate, sessile to subpetiolate, petioles 1.0–6.0 mm long, resupinate; leaves shape variable on the same individual, ovate to widely lanceolate, obovate, oblong, elliptic or rarely rotund, 2.0–5.5 × 4.0–12.0 cm, apex obtuse to rounded, truncate to subemarginate with a small mucron, base attenuate, margin hyaline; glabrate, glaucous, crasso–coriaceous, pinnate venation, middle vein fading; leaves prehensile when young. ***Inflorescences*** racemes, 1(2) per axil, indeterminate, 2.0–6.2 cm long, peduncle 0.5–1.0 cm long, rachis subterete, glabrous, green; 6–8(10) triads, opposite to subopposite, decussate, peduncle triad 0.6–2.0 mm long; bracts and bracteoles cymbiform, deciduous in anthesis. ***Flower buds*** cylindric to widely clavate with a rounded apex, 5.0–6.2 mm long. ***Mature flowers*** bisexual, sessile, hexamerous, 2.2–3.5 × 6.0–8.0 mm; petals linear, reflexed in the middle part to 45° up to 90°, 0.8–1.0 × 4.2–7.0 mm, white greenish to yellowish, petal apex rarely brown to reddish. ***Androecium*** with dimorphic stamens in two series, alternate, adnate to petals, filament free on middle part, papillate; first series with a filament 0.5–1.0 mm long, second series 0.3–0.6 mm long; symmetric anthers, dorsifixed, connective apiculate, anthers of the second series with a prominent connective; trichomes (villous) on the abaxial side of the anther up to 1.0 mm. ***Gynoecium*** with a convolute style, 3.2–5.0 mm long, 2(3) longitudinal folds, stigma capitate, slightly oblique; ovary inferior, cylindric 1.4–1.8 × 1.5–2.0 mm; nectary or nectariferous ring with 6 protuberances, globose, around the style; calyculus green–yellowish, irregular to dentate margin. ***Fruit*** a single-seeded berry, ovoid to subglobose, 4.6–7.5 × 7.8–12.0 mm, granulated, rusty; pedicel accrescent on the fruit, granulated. ***Seeds*** ovoid, 4.0–6.0 × 6.5–10.0 mm (Figs 2, 3).

##### Distribution, habitat, and hosts.

*Struthanthusvenetus* is known from oak and pine-oak forests, tropical deciduous and subdeciduous forests, urban areas, and crop fields. It occurs in central Mexico, mainly in the states of Morelos, Ciudad de México, Estado de México, Guanajuato, Guerrero, and Puebla (Fig. 5), at elevations from 850 to 2,445 m. It parasitizes species of at least 26 families, including gymnosperms and angiosperms: Adoxaceae E. Mey., Asteraceae Bercht. & J. Presl, Bignoniaceae Juss., Burseraceae Kunth, Casuarinaceae R. Br., Convolvulaceae Juss., Cupressaceae Gray, Fabaceae Lindl., Fagaceae Dumort., Meliaceae Juss., Moraceae Gaudich., Myrtaceae Juss., Oleaceae Hoffmanns. & Link, Opiliaceae Valeton, Petiveriaceae C. Agardh, Pinaceae Spreng. ex Rudolphi, Proteaceae Juss., Punicaceae Bercht. & J. Presl, Rosaceae Juss., Rubiaceae Juss, Rutaceae Juss, Salicaceae Mirb., Tiliaceae Juss., and Verbenaceae J. St.-Hil. Host records include other genera of parasitic plants such as *Phoradendron* (Santalaceae) and *Schoepfia* (Schoepfiaceae).

##### Phenology.

Flowering from April to November and with fruits from June to April.

##### Notes.

Older individuals have larger leaves near the base of the stem, leaf shape and size of variable size thorough the whole plant, and thick and corky petioles. Also, petals of some flower buds become reddish near the apex.

##### Additional specimens examined.

Mexico. **Ciudad de México** • Coyoacán, Universidad Nacional Autónoma de México (UNAM, between Institute of Biomedical Research and Sports Complex Buildings, 19.32437, -99.190957, 2229 m a.s.l., 22 Feb 2018 (fr), *M.A. Caraballo 3383* (US) • Cuauhtémoc, alameda central a 10 m de la avenida Hidalgo. Colonia Centro, 19°26'08.2"N, 99°08'42.65"W [19.435561, -99.14518], s.d., *L. Agonizante N. s.n.* (UAMIZ) • ibid., San Antonio Abad y avenida del Taller, col. Tránsito, 19°24'50.61"N, 99°08'4.66"W, 2,250 m a.s.l., 23 Feb 1974 (fr), *J. Gimate L. s.n.* (ENCB) • Iztapalapa, dentro del campus de la UAM-Iztapalapa, calle San Rafael Atlixco, 19°21'36.8"N, 99°04'17.8"W, 2,240 m a.s.l., 4 Aug 2022 (fl), *R. Cerros T. s.n.* (HUMO) • Gustavo A. Madero, Sierravista 353, jardinera frente a tienda. En Lindavista, 19°29'55.25"N, 99°08'42.65"W [19.49868, -99.12861], 2,240 m a.s.l., 15 Oct 2023 (fl, fr), *M.G. Maldonado B. 183* (HUMO). **Estado de México** • Ixtapan de la Sal, 18°50'31.49"N, 99°40'48.11"W, 1,876 m a.s.l., 15 May 2023 (fl), *M.I. Miguel V. 1305* (HUMO) • ibid., 18°50'53.45"N, 99°40'52.27"W, 1,913 m a.s.l., 15 May 2023 (fl), *M.I. Miguel V. 1306* (HUMO) • Temascaltepec, Chorrera [Carnicería], 19°02'40"N, 100°01'19.5"W, 1,230 m a.s.l., 8 Aug 1932 (fl), *G.B. Hinton 1281* (DES, GH, US) • Tejupilco, en el cerro de la Muñeca, 18°54'40"N, 100°08'20"W, 1,500 m a.s.l., 27–28 Feb 1954 (fr), *E. Matuda 30519* (MEXU) • Tepetlixpa, carretera Atlatlahucan-Tepetlixpa, 1 km antes de la desviación a Nepantla, 18°58'47.87"N, 98°51'10.81"W [18.979963, -98.853003], 1,964 m a.s.l., 2 Aug 2022 (fl), *M.I. Miguel V. 1027* (HUMO) • Tonatico, salida de Tonatico (N-S), 18°48'22.68"N, 99°39'52.27"W, 1,668 m a.s.l., 15 May 2023 (fl), *M.I. Miguel V. 1307* (HUMO). **Guanajuato** • León de los Aldama, blvd. Adolfo López Mateos, en barranca frente a Mobil 1, cerca del parque los Cárcamos, 21°09'49.32"N, 101°41'3.44"W, 1,829 m a.s.l., 5 Jul 2023 (fl), *M.G. Maldonado B. 142* (HUMO) • ibid., en parque Cárcamos, Av. Adolfo López Mateos, esquina con blvd. José María Morelos, en árboles frente al lago, 21°10'3.14"N, 101°41'3.88"W, 1,822 m a.s.l., 5 Jul 2023 (fl), *M.G. Maldonado B. 143* (HUMO) • ibid., en parque Cárcamos, av. Adolfo López Mateos esquina con blvd. José María Morelos, en árboles frente al lago, 21°10'2.89"N, 101°40'59.99"W, 1,823 m a.s.l., 5 Jul 2023 (fl), *M.G. Maldonado B. 144* (HUMO). **Guerrero** • Chilpancingo de los Bravo, col. Juquila, en terreno baldío en la calle Cuauhtémoc, esquina con Miguel Hidalgo, 17°31'52.20"N, 99°30'40.82"W, 1,337 m a.s.l., 15 Jan 2023 (fr), *M.G. Maldonado B. 83* (HUMO) • ibid., 15 Jan 2023 (fl), *M.G. Maldonado B. 84* (HUMO)• ibid., 15 Jan 2023 (fl), *M.G. Maldonado B. 85* (HUMO) • ibid., 27 Feb 2023 (fl), *M.G. Maldonado B. 86* (HUMO) • Leonardo Bravo, Corral de Piedra, a lado del tecorral que delimita el patio trasero del sr. Hipólito Maldonado, 17°39'45.01"N, 99°56'36.35"W, 1,563 m a.s.l., 23 Apr 2023 (fl, fr), *M.G. Maldonado B. 95* (HUMO) • Pilcaya, el Mogote, 18°40'55.31"N, 99°33'57.27"W, 1,504 m a.s.l., 15 May 2023 (fl), *M.I. Miguel V. 1303* (HUMO) • ibid., loc. Piedras Negras, costado de la carretera, 18°43'23.59"N, 99°36'30.28"W, 1,557 m a.s.l., 15 May 2023 (fl), *M.I. Miguel V. 1304* (HUMO) • Taxco de Alarcón, Axixintla; km 194 carretera Taxco-Cuernavaca, 18°37'43.9"N, 99°30'39.9"W, 1,940 m a.s.l., 13 May 2021 (fl, fr), *M.G. Maldonado B. 47* (HUMO) • ibid., Palmillas; km 29 carretera Taxco-Cuernavaca, 18°30'52.6"N, 99°25'35.3"W, 1,940 m a.s.l.,13 May 2021 (fl, fr), *M.G. Maldonado B. 48* (HUMO). **Morelos** • Amacuzac, km 18 de la carretera de cuota a Acapulco, a 2 km de Casahuatlán, 100 m a orilla de carretera, 18°33'57"N, 99°24'27"W, 987 m a.s.l., 3 Jul 2007 (fl, fr), *L.G. Galván-González 123* (MEXU, UAMIZ, IEB) • Atlatlahucan, carretera Cuautla-Amecameca, 10 m adelante de la desviación a Yecapixtla, a un costado de la carretera, 18°53'52.61"N, 98°54'29.13"W, 1,518 m a.s.l., 2 Aug 2022 (fl), *M.I. Miguel V. 1026* (HUMO) • ibid., carretera Atlatlahucan-Totolapan, en la esquina con la calle Galeana, a un costado de la carretera, 18°56'44.9"N, 98°53'52.3"W, 1,680 m a.sl., 2 Aug 2022 (fl), *M.I. Miguel V. 1028* (HUMO) • Coatlán del Río, rumbo al parque ecoturístico el Hoyanco, a orillas del canal de agua, 18°43'21"N, 99°25'51"W, 1,219 m a.s.l., 24 Feb 2013 (fr), *L.G. Galván-González 212* (HUMO) • Cuautla, 420 m antes de la empresa Campi, en dirección de sur a norte, a un costado de la carretera Izúcar de Matamoros–Cuautla, 18°50'32.78"N, 98°55'37.56"W, 1,367 m a.s.l., 30 May 2023 (fl), *M.I. Miguel V. 1309* (HUMO) • Cuernavaca, on shrubs, hillsides above Cuernavaca, 9 Nov 1895 (fl), *C.G. Pringle 6185* (MEXU; UC; HUH; US; P; VT; COLO) • ibid., near Cuernavaca, 2 Jun 1939 (fl), *O. Nagel 8026* (GH, MEXU) • ibid., about 4.5 mi E of Ocotepec on road from Cuernavaca to Tepoztlán, [18°58'39.85"N, 99°09'39.85"W, 1,643 m a.s.l.], 4 Aug 1971 (fl), *W.D. Stevens 1385* (ENCB) • ibid., Colonia del Bosque, 18°54'49.4"N, 99°15'17.1"W, 1,510 m a.s.l., 31 May 2014 (fr), *N.R. Rueda 2* (HUMO) • ibid., en barranca de la col. la Unión, 18°54'49.4"N, 99°15'17.1"W, 1,490 m a.s.l., 31 May 2014 (fl), *L.G. Galván-González 221* (HUMO) • ibid., Facultad de Ciencias del Deporte, campus Chamilpa de la UAEM, 18°59'7.03"N, 99°14'18.46"W, 1,932 m a.s.l., 21 Nov 2016 (fl, fr), *L.G. Galván-González 367* (HUMO) • ibid., la Carpa brecha a la barranca del Túnel, 18°57'2.99"N, 99°14'29.92"W, 1,908 m a.s.l., 5 Jul 2021 (fl), *M.I. Miguel V. 964* (HUMO) • ibid., jardín botánico de la UAEM, 18°59'11.4"N, 99°17'15.3"W, 1,906 m a.s.l., 18 May 2022 (fl, fr), *M.G. Maldonado B. 60* (HUMO) • ibid., jardín etnobotánico Acapatzingo, calle Mariano Matamoros esquina con Degollado, 18°54'27.91"N, 99°13'29.951"W, 1,462 m a.s.l., 8 May 2023 (fl), *M.I. Miguel V. 1300* (HUMO) • Emiliano Zapata, Tetecalita, entrando por vv. Las Granjas, 18°46'26.7"N, 99°10'0.2"W, 1,194 m a.s.l., 18 Dec 2014 (fr), *Y. Montoya M. 369* (HUMO) • Jantetelco, faldas del cerro del Chumil, 18°42'42"N, 98°45'16"W, 1,428 m a.s.l., 1 Sep 2006 (fl, fr), *L.G. Galván-González 15* (HUMO) • ibid., Chalcatzingo zona arqueológica, 18°40'40.38"N, 98°46'14.18"W, 1,365 m a.s.l., 9 May 2023 (fl), *M.I. Miguel V. 1301* (HUMO) • ibid., desviación junto al puente Jantetelco, en la carretera Puebla-Jantetelco, frente al parque, 18°42'58.54"N, 98°46'1.1"W, 1,435 m a.s.l., 19 Jun 2023 (fl), *M.G. Maldonado B. 123* (HUMO) • Jiutepec, parque estatal el Texcal, 250 m al noreste de la entrada principal, cerca del borde de la reserva, a un costado de una brecha, 18°53'46.79"N, 99°08'26.12"W, 1,408 m a.s.l., 31 May 2023 (fl), *M.I. Miguel V. 1324* (HUMO) • Jonacatepec, 20 m antes de la planta tratadora de agua, a un costado de la carretera Tepalcingo-Jonacatepec, 18°39'41.09"N, 98°48'37.88"W, 1,295 m a.s.l., 30 May 2023 (fl), *M.I. Miguel V. 1308* (HUMO) • Miacatlán, Xochicalco, 1.85 km al S de la zona arqueológica, 18°47'35.4"N, 99°17'48.53"W, 1,198 m a.s.l., 10 May 2023 (fl), *M.I. Miguel V. 1302* (HUMO) • Puente de Ixtla, Tilzapotla. El Espinazo del Diablo, 1.5 km al este de Tilzapotla, brecha de terracería mina la Parreña, entronque con la Piaña-carretera el Salto, [18°29'31.66"N, 99°14'25.92"W, 1,410 m a.s.l.], 30 Jun 1998 (fl), *J.C. Juárez 993* (HUMO) • ibid., carretera Tilzapotla-Caxintla, 300 m pasando la cantera, 1.5 km antes de la desviación hacia el Salto, 18°28'51.7"N, 99°15'50"W, 1,037 m a.s.l., 9 Feb 2023 (fr), *M.I. Miguel V. 1251* (HUMO) • Temixco, barranca los Sabinos, 18°48'35"N, 99°15'58"W, 1,215 m a.s.l., 10 Nov 2006 (fl buds), *R. Cerros T. 2725* (UAMIZ) • Tepoztlán, carretera Tepoztlán-Oacalco, 18°58'10"N, 99°04'25"W, 1,560 m a.s.l., 28 Sep 2006 (fr), *R. Cerros 2676* (CIIDIR, MEXU, UAMIZ) • ibid., Cerro de la Cruz, cerca de San Andrés de la Cal. [18°57'35.73"N, 99°6'59.76"W, 1,200 m a.s.l.], 4 Apr 2009 (fl), *M. Cházaro B. 9401* (XAL) • ibid., alrededor del poblado de San Andrés de la Cal, 18°57'09"N, 99°06'36.1"W, 1,783 m a.s.l., 7 Jun 2020 (fl), *R. Hernández C. 2439* (HUMO) • Tlaltizapán, balneario Sta. Isabel, área de acampado, 18°43'58.44"N, 99°06'51.22"W, 954 m a.s.l., 15 Jun 2023 (fl), *M.I. Miguel V. 1346* (HUMO) • Yautepec, huerta de una casa particular (la familia Chávez) a ca. 1 km al NE de Yautepec [Tabachines], 18°53'55.6"N, 99°02'16.9"W, 1,231 m a.s.l., 4 Apr 1986 (fr), *D.H. Lorence 5033* (RSA, US) • ibid., carretera Tlayacapan-Oaxtepec, 400 m adelante de la desviación a Atlatlahucan, 18°55'39.15"N, 98°57'59.59"W, 1,481 m a.s.l., 2 Aug 2022 (fl remains), *M.I. Miguel V. 1029* (HUMO) • ibid., 200 m adelante de la excaseta de Oacalco, autopista Oaxtepec-Tepoztlán, 18°55'59.23"N, 99°01'39.22"W, 1,367 m a.s.l., 30 May 2023 (fl), *M.I. Miguel V. 1310* (HUMO) • Yecapixtla, Yecapixtla, [18°52'9.13"N, 98°49'47.98"W], 1,700 m a.s.l., 10 Oct 2014 (fl), *L. Ortega C. 148* (HUMO) • Zacualpan de Amilpas, Campos de cultivo de Tlacotepec, 18°48'46"N, 98°44'48"W, 1,770 m a.s.l., 22 Sep 2006 (fr), *L.G. Galván-González 44* (UAMIZ). **Puebla** • Atlixco, camino Puebla-Atlixco, 200 m después de la caseta de cobro Siglo XXI, 18°56'09.01"N, 98°26'58.31"W, 1,927 m a.s.l., 19 Jun 2021 (fl, fr), *M.G. Maldonado B. 122* (HUMO) • Puebla, jardín botánico de la BUAP, 18°59'57.51"N, 98°11'49.65"W, 2,150 m a.s.l., 12 Dec 2017 (fr), *A.J. Coombes 1808* (HUAP) • Puebla, camellón frente al jardín botánico de la BUAP, 18°59'57.6"N, 98°11'53.15"W, 2,132 m a.s.l., 30 Sep 2022 (fl, fr), *M.I. Miguel V. 1032* (HUMO) • ibid., camellones de la BUAP, 18°59'56"N, 98°11'50.06"W, 2,134 m a.s.l., 9 Jun 2023 (fl), *M.I. Miguel V. 1345b* (HUMO) • Tepexco, al N de loc. de Ixtlala, 18°35'37.58"N, 98°42'8.3"W, 1,151 m a.s.l., 24 Aug 2016 (fr), *L. Caamaño O. 9052* (HUAP, HUMO) • 1.5 km al N del balneario Ixtatlala, sobre la brecha al poblado de Tepexco, 18°36'16.2"N, 98°42'20.2"W, 1,154 m a.s.l., 28 Aug 2022 (fl), *M.I. Miguel V. 1030* (HUMO) • ibid., 28 Aug 2022 (fl, fr), *M.I. Miguel V. 1031* (HUMO).

## ﻿Discussion

The findings obtained in this study support the idea that *S.venetus* should be reinstated and no longer be considered a synonym of *S.interruptus*. The unique combination of bisexual flowers, granular fruits, and crasso–coriaceous leaves help to recognize this taxon from other Mexican species of *Struthanthus*. Other characters that can help separate *S.venetus* from *S.interruptus* include young branches with glaucous stems that become brown to brown-grayish when mature (vs. young branches green with brown to reddish-brown stems, and glossy with lenticels when mature in *S.interruptus*); leaf petioles that are subsessile to decurrent and 0.1–0.6 cm long (vs. leaf petioles decurrent and 0.6–1.0 (1.5) cm long in *S.interruptus*); and bisexual flowers with accrescent pedicels bearing granulated fruit ripening rusty red (vs. unisexual flowers with non-accrescent pedicels bearing smooth fruits ripening reddish in *S.interruptus*; Fig. 3C, D). In addition, the distributional range recorded for *S.venetus* does not overlap with the one known for *S.interruptus* (Fig. 6).

*Struthanthusvenetus* was described based on a fruiting individual. Similarly, *S.interruptus* was collected with pistillate flowers (no longer extant in the specimen) and unripe fruits, and its diagnosis remarked that male flowers and fruit were insufficiently known (Fig. 5A, C). Incomplete specimens have led to numerous synonyms in *Struthanthus* through the description of new taxa that are subsequently merged (Caires and Dettke 2020). It is noteworthy to mention that proper identification of specimens in *Struthanthus* is very difficult in the absence of material containing both staminate and pistillate flowers (for dioecious species) and fruit. Our results from the pollen germination test provide evidence that pollen of *S.venetus* can germinate and develop pollinic tubes of considerable length. Additional studies on pollen germination in *Struthanthus* can help to understand better the reproductive mechanisms of fertilization, especially for species with convolute styles. We did not conduct pollen viability tests in *S.interruptus* because we considered that differences in flower morphology with *S.venetus* (dioecious vs. bisexual flowers) were enough evidence that both taxa represent separate biological entities. It is worthy to note that none of the species of *Struthanthus* from México have been the subject of studies on pollen germination. Only *S.flexicaulis* Mart. and *S.vulgaris* (Vell.) Mart. (both from Sao Paulo, Brazil) have been the subject of embryological studies (Venturelli 1981, 1984).

The study by Kuijt in 1975 showed that *S.venetus* has been misidentified as *S.interruptus*, *S.condensatus*, and *S.crassipes*. However, *S.venetus* can be separated from all these taxa by its distinctive bisexual flowers. Also, *S.venetus* has a racemose inflorescence with free bracts and deciduous bracteoles that contrast with the capitate inflorescence with fused bracts and bracteoles that persist after anthesis seen in *S.condensatus*. Additionally, *S.venetus* bear one or rarely two racemes per axil, leaves with obtuse to rounded, truncate or subemarginate apex and attenuated base, and granulated fruiting pedicels. These features diverge from the ones found in *S.crassipes*, which has one to three (rarely five) racemes per axil, leaves with attenuate to acuminate apex and obtuse to round base, and smooth fruiting pedicels (Table 1).

**Table 1.** Morphological differences among *Struthanthuscondensatus*, *S.crassipes*, *S.interruptus*, and *S.venetus* (Loranthaceae).

Characters	*S.condensatus*	*S.interruptus*	*S.crassipes*	*S.venetus*
Plant sexuality	dioecious	dioecious	dioecious	monoecious-monocline
Leaf shape	lanceolate, ovate to rounded, rarely elliptic	oblong to ovate-elliptical, rarely obovate or rounded	lanceolate to elliptic-lanceolate	ovate to widely lanceolate, obovate, oblong, elliptic, rarely rounded
Leaf apex	obtuse to rounded	obtuse to rounded, rarely truncate or acute	attenuate to acuminate	obtuse to rounded, truncate to subemarginate with a small mucro
Leaf base	obtuse to rounded	decurrent	obtuse to rounded	attenuate
Leaf size (cm)	2.0–5.0 × 4.0–7.2	1.5–5.0 × 5.5–10.2	1.8–5.5 × 5.5–12.5 (13.5)	2.0–5.5 × 4.0–12.0
Leaf texture	coriaceous to crasso–coriaceous	papery or chartaceous	coriaceous	crasso–coriaceous
Petiole length (cm)	0.2–0.6	0.6–1.0 (1.5)	0.3–1.2	0.1–0.6
Inflorescences type	capitate	spiciform	racemose	racemose
Inflorescences per axil	1	1–2	1–3 (5)	1 (2)
Triads per inflorescence	2–6	8–12 (14)	6–16	6–8 (10)
Peduncle triad length (mm)	0	0	4.0–10.0	0.6–2.0
Bracts and bracteoles	fused into a cup, persistent	not fused, deciduous	not fused, bracteoles persistent in anthesis	not fused, deciduous
Flower length from base of ovary (mm)	3.5–5.5	4.0–7.5	4.0–7.0	6.0–8.0
Fruit pedicel	not applicable (fruit sessile)	accrescent, smooth	evidently accrescent, pendent and smooth	accrescent, granulated
Fruit shape	ovoid to ellipsoid	ellipsoid	ovoid to obovoid	ovoid to subglobose
Fruit size (mm)	4.0–6.0 × 5.0–7.5	6.5–7.0 × 10.8–12.0	3.8–6.0 × 6.5–8.6	4.0–6.0 × 6.5–10.0
Fruit surface	smooth	smooth	smooth	granulated
Color of ripe fruit	maroon	reddish	orange-reddish to rusty	rusty

*Struthanthusvenetus* has a spotty distribution with the core found in central México at the states of Ciudad de México, Estado de México, Guerrero, Morelos, and Puebla. There are also a handful of disjunct specimens from León de los Aldama in Guanajuato, about 280 km away from the westernmost edge of the main distribution in the Estado de México (Fig. 6). These collections were made on ornamental plants in public parks, and it is possible that these ornamental trees originated from Morelos or the Estado de México, given that these states are the main producers of ornamental plants for the country (SIAP 2020).

The taxonomy of *Struthanthus* and related genera needs to be investigated from an interdisciplinary perspective, and future work with expanded sampling covering *Struthanthus* and related genera should help clarify their taxonomy and evolutionary history. These future studies should incorporate a strong component of fieldwork, given that examining living plants is very important to detect morphological variation and recognize overlooked taxa, especially when type specimens are incomplete and/or severely fragmented. Finally, we would like to mention that in this study we did not address directly the taxonomy of *S.interruptus* because our surveys indicate that, as currently defined, it seems to involve at least three more overlooked taxa. Therefore, we decided to discuss the taxonomy of *S.venetus* by itself and address the rest of the *S.interruptus* complex in a separate publication.

## Supplementary Material

XML Treatment for Struthanthus venetus

## ﻿Additional information

### Author ORCIDs

Maria Guadalupe Maldonado-Borja https://orcid.org/0000-0002-9515-6969

Mónica Isabel Miguel-Vázquez https://orcid.org/0000-0003-1418-8422

Adolfo Espejo-Serna https://orcid.org/0000-0001-7192-4612

Rosa Cerros-Tlatilpa https://orcid.org/0000-0002-7079-6230

### Data availability

All of the data that support the findings of this study are available in the main text.
